# Preeclampsia pathophysiology and adverse outcomes during pregnancy and postpartum

**DOI:** 10.3389/fmed.2023.1144170

**Published:** 2023-03-16

**Authors:** Courtney Bisson, Sydney Dautel, Easha Patel, Sunitha Suresh, Patricia Dauer, Sarosh Rana

**Affiliations:** Department of Obstetrics and Gynecology, NorthShore University Health System, Evanston, IL, United States

**Keywords:** preeclampsia, long-term effect, pregnancy, hypertension, morbidity

## Abstract

**Background:**

Preeclampsia is a disease with far-reaching consequences that extend beyond the immediate postpartum period and have a significant impact later in life. Preeclampsia exerts an effect on most organ systems in the body. These sequelae are mediated in part by the incompletely elucidated pathophysiology of preeclampsia and the associated vascular changes.

**Content:**

Current research focuses on unraveling the pathophysiology of preeclampsia with the goal of implementing accurate screening and treatment modalities based on disease development and progression. Preeclampsia causes significant short- and long-term maternal morbidity and mortality, not only in the cardiovascular system but also in other organ systems throughout the body. This impact persists beyond pregnancy and the immediate postpartum period.

**Summary:**

The goal of this review is to discuss the current understanding of the pathophysiology of preeclampsia as it relates to the adverse health consequences in patients impacted by this disease, along with a brief discussion of ways to improve overall outcomes.

## 1. Background and epidemiology

Hypertensive disorders of pregnancy (HDP) represent a major cause of pregnancy-associated morbidity and mortality. These disorders have far-reaching consequences that extend well beyond the pregnancy and the immediate postpartum period. According to the Centers for Disease Control (CDC), HDP, including preeclampsia, accounts for nearly 7% of all maternal deaths (1). HDP consists of a myriad of diagnoses, including chronic hypertension, gestational hypertension, preeclampsia, preeclampsia with severe features, and eclampsia (2, 3). Similar to chronic hypertension, data suggest that preeclampsia has significant sequelae later in life. Therefore, a thorough understanding of the pathogenesis and prediction of HDP and its implications on short- and long-term health outcomes is crucial to provide optimal care to pregnant patients, especially as they transition out of the immediate postpartum period.

## 2. Risks and diagnosis

To improve long-term morbidity in the pregnant patient, minimizing the development of disease and early detection are paramount. No perfect prediction model exists to identify all patients who will develop HDP accurately nor define those at greatest risk of long-term morbidity. However, risk factors for the disease have been identified. As determined by the American College of Obstetrics and Gynecology (ACOG), risk factors for the development of preeclampsia include prior preeclampsia, chronic hypertension, diabetes, renal disease, autoimmune diseases such as lupus, and multifetal gestations (2, 4, 5). Traditionally, the Black race has been identified as a risk factor for the development of HDP, with Black people having much higher rates of preeclampsia compared to White counterparts (6). Recent research into health inequities, however, has questioned whether one’s race or ethnicity is a concrete risk factor for HDP or whether race and ethnicity are merely reflective of unequal access to care and unfavorable socio-economic conditions present in the healthcare system and society.

### 2.1. Diagnosis

Preeclampsia and HDP have well-established guidelines that aid clinicians in diagnosis (Table 1). (2, 7) Chronic hypertension is defined as a diagnosis that predates the pregnancy or blood pressure elevations (≥ 140/90) diagnosed prior to 20 weeks on two occasions at least 4 h apart (8). Though these patients have a diagnosis of chronic hypertension prior to pregnancy, they remain at risk of worsening hypertension and the development of preeclampsia. Gestational hypertension is diagnosed with two elevated blood pressure readings ≥140/90 (defined as mild range blood pressures) after 20 weeks on two occasions at least 4 h apart (2). Patients with this diagnosis lack the overt signs and symptoms of preeclampsia, though they are at increased risk of developing preeclampsia. Patients diagnosed earlier in pregnancy have a higher risk of progression to preeclampsia, with 15–25% of patients ultimately developing preeclampsia (9). This risk of progression increases the earlier a patient is diagnosed and necessitates close patient monitoring.

**Table 1:** Diagnostic criteria for HDP.

HDP	Blood pressure criteria	Lab values	Signs and symptoms
Gestational hypertension	BP≥140 or ≥90, >4 h apart after 20 weeks gestation	N/A	N/A
Preeclampsia without severe features	BP ≥140 or ≥90, >4 h apart after 20 weeks gestation	Proteinuria defined as: At least 300 mg in 24-h urine OR Protein/creatinine ratio ≥ 0.3	N/A
Preeclampsia with severe features	BP ≥160 or ≥110, >4 h apart after 20 weeks gestation OR BP ≥160 or ≥ 110 after 20 weeks gestation requiring acute treatment OR BP ≥140 or ≥ 90 with lab and/or symptom criteria	Thrombocytopenia (platelets <100×10^9/L) AND/OR Transaminitis (AST/ALT twice normal) AND/OR Acute kidney injury (doubling of patient’s baseline creatinine OR > 1.1)	Intractable headache Persistent vision changes Severe right upper quadrant pain Pulmonary edema
HELLP syndrome (hemolysis, elevated liver enzymes, low platelets)	BP ≥140 or ≥ 90 with lab and symptom criteria* *About 15% of patients HELLP syndrome may not have hypertension	Thrombocytopenia (platelets <100×10^9/L) AND Transaminitis (AST/ALT twice normal) AND Hemolysis LDH > 600 U/l Haptoglobin <25 mg/dl Bilirubin ≥1.2 mg/dl Schistocytes on peripheral smear	Variable
Eclampsia	Variable	Variable	Seizures

Adapted from Ref. (2).

Preeclampsia itself is defined as newly elevated blood pressure ≥ 140/90 after 20 weeks’ gestation in addition to proteinuria defined as 300 mg or more in a 24-h urine specimen, or a protein/creatinine ratio of 0.3 or more, or 2+ protein on urine dipstick (used if other quantitative methods are unavailable) (2). A patient meeting these criteria is diagnosed with preeclampsia without severe features. It is important to recognize that there are other ways to meet the criteria for preeclampsia, all of which upstage the disease process to preeclampsia with severe features. Differentiating these two entities is important for management decisions and pregnancy implications. Preeclampsia with severe features represents a more severe form of the disease and has various diagnostic criteria, which can generally be divided into three main categories: blood pressure values (severe range blood pressures defined as ≥160/110), laboratory values, and symptomatology. Patients with blood pressure elevations of ≥160/110 (with *either* systolic *or* diastolic elevations) with two readings at least 4 h apart or continuous severe range blood pressures necessitating rapid treatment are formally diagnosed with preeclampsia with severe features (2). Even without severe range blood pressures and only mild range blood pressures, patients with the following laboratory criteria are diagnosed with preeclampsia with severe features: thrombocytopenia (platelets <100×10^9/L), renal insufficiency (doubling of patient’s baseline creatinine or creatinine >1.1 mg/dl), or liver impairment (liver enzymes twice the normal value) (2, 7, 10). Lastly, patients with or without severe range blood pressures and symptoms of pulmonary edema, severe right upper quadrant pain not due to other etiologies, persistent vision changes, and new-onset headache refractory to medications may also be diagnosed with preeclampsia with severe features (2, 7, 10). Patients with pre-existing chronic hypertension may meet the criteria for superimposed preeclampsia with severe features if they exhibit any of the laboratory abnormalities or symptoms (2, 7, 11).

A more severe form of preeclampsia known as HELLP syndrome (hemolysis, elevated liver enzymes, and low platelets) presents increasing rates of mortality and adverse maternal and fetal outcomes (12). Patients demonstrate signs of hemolysis (elevated lactate dehydrogenase [LDH] > 600 IU/l), elevated liver enzymes (more than twice normal lab values), and low platelets (<100×10^9/L) (2, 13). Finally, eclampsia represents the most severe form of the disease and is categorized by new-onset seizures, typically in patients who already carry a diagnosis of preeclampsia (2). Importantly 20–30% of patients diagnosed with eclampsia will be normotensive or have no other disease manifestation, so a high index of suspicion is crucial (14, 15).

Several diagnostic conundrums exist regarding the accurate diagnosis of preeclampsia, most notably in the setting of superimposed disease and patient symptoms. Patients with chronic hypertension may have worsening of their blood pressure during pregnancy, though this does not inherently warrant a diagnosis of preeclampsia. Patient observation and evaluation are required to characterize the diagnosis in this patient subset further. Additionally, patients that meet preeclampsia criteria with persistent symptoms represent a unique cohort. These symptoms require a full workup, and evaluation is vital to rule out other causes of symptomatology as preeclampsia symptoms are vague and non-specific (16).

### 2.2. Clinical manifestations

Patients with preeclampsia generally present with some degree of blood pressure elevation (17). They may have an unrelenting headache, right upper quadrant pain, or vision changes. Occasionally, patients may complain of increased lower extremity edema, which, while not diagnostic of preeclampsia, should certainly raise concern for disease development. In the absence of symptoms, laboratories drawn for any indication that could be indicative of developing preeclampsia should alert the clinician’s suspicion. Patients generally have their blood pressure checked at every appointment, and the overall blood pressure trend is important as patients who ultimately are diagnosed with preeclampsia often have increased blood pressure during their pregnancy (18). In addition to patient symptoms, the fetus can often have pathology consistent with preeclampsia. While not specified in the guidelines in the United States by the American College of Obstetrics and Gynecology (ACOG), international organizations recommend including fetal growth restriction and other signs of uteroplacental insufficiency in the diagnosis of preeclampsia (7). Any change in the fetal growth or well-being should therefore prompt a thorough evaluation by the clinician.

Though a more thorough discussion of diagnostic modalities and criteria is beyond the scope of this article, recognizing signs and symptoms of HDP is important as those diagnosed earlier in pregnancy have a higher risk of progression to more severe disease and possibly a higher risk of long-term morbidity (9).

## 3. Pathophysiology

**Figure 1:**
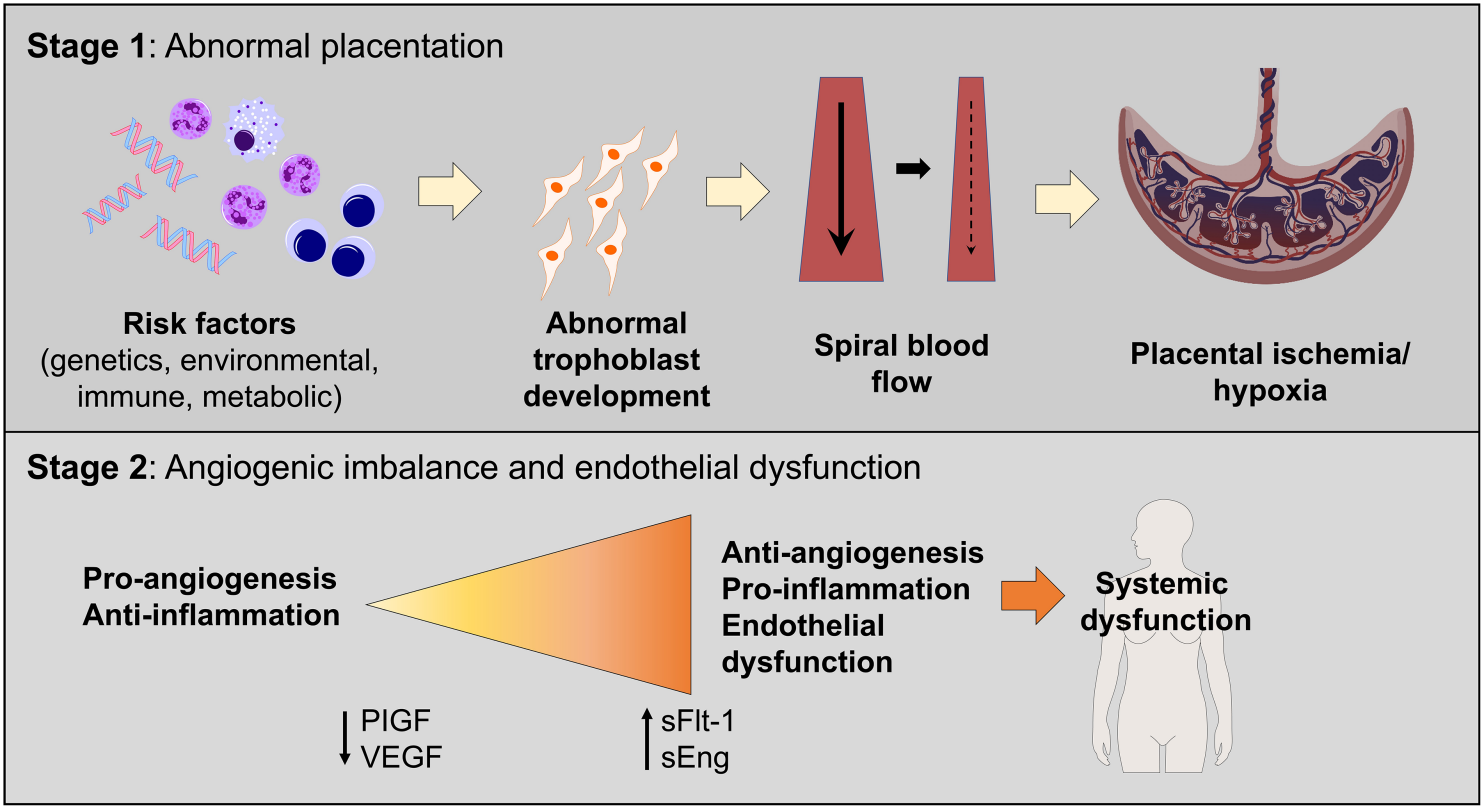
Two-staged model of preeclampsia pathogenesis. Stage 1 consists of the preclinical stage and is characterized by abnormal placentation, which leads to the release of soluble factors in maternal circulation, leading to systemic endothelial dysfunction and hypertension (Stage 2).

Although the pathophysiology of HDP is not fully understood, proposed contributors include placental dysfunction and immunologic changes culminating in poor uteroplacental perfusion (Figure 1). Importantly, the underlying mechanisms thought to contribute to vascular dysfunction in preeclampsia are like those in cardiovascular and atherosclerotic diseases in the non-pregnant individual. These similarities may help explain why preeclampsia is associated with an increased risk of cardiovascular disease later in life.

In normal pregnancy, cytotrophoblasts invade the uterine myometrium and spiral arteries to create a rich network of vascular anastomoses that will ultimately perfuse the placenta and fetus (19). In patients with preeclampsia, cytotrophoblasts do not develop the invasive phenotype required to create these robust anastomoses, which leads to decreased and shallow endovascular invasion of the spiral arteries (20–22). These abnormal blood vessels have narrow caliber, which leads to placental ischemia and ineffective oxygen transfer (23). This is demonstrated in the Stage 1 portion of Figure 1. Additionally, higher levels of various pro-inflammatory molecules are noted in patients with preeclampsia, including natural killer cells and other non-specific markers of inflammation (5, 24). In a normal pregnancy, an “immune tolerance” exists, largely due to changes in the maternal immune system surrounding T cells (24). In pregnancies not impacted by preeclampsia, Th1 cells and Th2 cells exist in harmony to prevent excessive inflammation and fetal rejection. In models of preeclampsia, this balance is disrupted, and many T cells shift to a Th1 phenotype, like those with chronic autoimmune diseases (19, 24). Th1 cells promote inflammation *via* pro-inflammatory cytokines, autoantibodies, and increased oxidative stress, which further worsens the damage and ischemia noted in preeclampsia (24).

The complex process of the development of preeclampsia may be facilitated by a combination of abnormal placentation and ischemia, which results in the release of pro-inflammatory and anti-angiogenic proteins in maternal circulation, ultimately resulting in endothelial dysfunction leading to the clinical syndrome seen in patients with preeclampsia. The two most studied and implicated biomarkers, especially in relation to the development of preeclampsia, are soluble FMS-like tyrosine kinase-1 (sFlt-1) and placental growth factor (PlGF) (5). sFlt-1 is an anti-angiogenic factor that inhibits neovascularization (25). Higher levels of sFlt-1 are found in patients with preeclampsia and the placentas of patients with preeclampsia (25, 26). The levels of PlGF are lower, and the ratio between sFlt-1 and PlGF is elevated in patients with preeclampsia (26, 27). This is demonstrated in the Stage 2 portion of Figure 1.

Overall, the pathogenesis of preeclampsia is extremely complex and likely multifactorial. The proposed main tenets in the development suggest abnormal placentation resulting in inappropriate spiral artery remodeling, and the resultant tissue hypoxia causes endothelial damage leading to hypertensive pathology. Meanwhile, changes in the maternal immune system in patients with preeclampsia facilitate a low level of chronic inflammation, which continues to perpetuate the cycle of endothelial damage. This combination may result in imbalances in angiogenic and anti-angiogenic factors. The complex interplay between placental pathology, inflammation, and changes in angiogenesis ultimately results in the clinical syndrome known as preeclampsia and contributes to adverse health outcomes in patients during pregnancy and postpartum (28, 29).

## 4. Adverse outcomes related to preeclampsia

**Figure 2:**
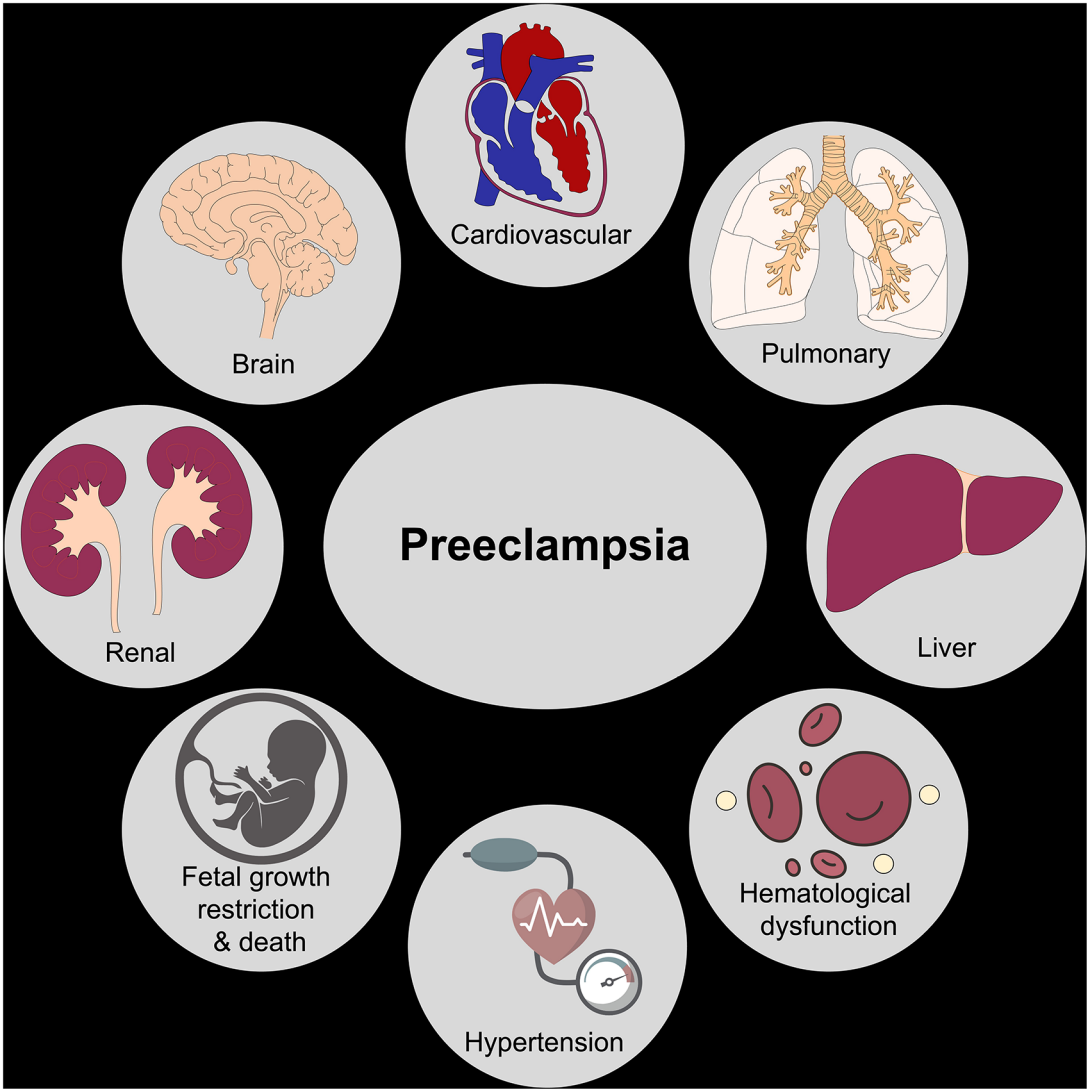
Organ systems impacted by preeclampsia. The figure shows various organ systems affected by preeclampsia leading to short-term and long-term maternal/fetal morbidity and mortality.

Preeclampsia can result in significant health impairment in patients during pregnancy, immediately postpartum, and beyond. Understanding the risks of preeclampsia and the pathophysiology of the disease process can aid in the prevention of maternal morbidity and mortality. The spectrum of these adverse outcomes is discussed in detail below and illustrated in Figure 2.

### 4.1. Maternal adverse outcomes associated with HDP

The development of preeclampsia profoundly impacts the cardiovascular system. In the short term, severe cases of preeclampsia can lead to cardiac dysfunction and severe hypertension and are also associated with peripartum cardiomyopathy (30, 31). Studies examining cardiac structure and function in preeclampsia have shown marked diastolic dysfunction and left ventricular remodeling beginning in pregnancy (32). Importantly, this type of remodeling is sometimes irreversible, and though it may be clinically apparent during pregnancy, it does not always regress in the postpartum setting (32). Patients with preeclampsia have a two-fold increase in the subsequent development of both fatal and non-fatal ischemic heart disease (33). These patients demonstrate accelerated cardiovascular aging, illustrating increased overall arterial stiffness like older, postmenopausal patients (34).

The risk of the development of heart failure over the first 5 years postpartum is elevated in patients with HDP, especially those with pre-existing chronic hypertension (35). Importantly, when controlling for other factors, non-Hispanic Black patients consistently had higher rates of heart failure following HDP compared to their non-Hispanic White counterparts (35). The development of HDP also significantly increases the risk of sustained hypertension in the postpartum period as well as over the course of a patient’s life, developing disease earlier in life than those without a preeclampsia history (25, 30, 31). A study in the United Kingdom investigating blood pressure changes in patients with preeclampsia diagnosed prior to 34 weeks illustrated an increase in immediate postpartum and long-term systolic and diastolic blood pressures over 13 years (36). This was in comparison to those diagnosed after 34 weeks, indicating that earlier preeclampsia diagnosis is correlated with worse long-term blood pressure parameters. Studies such as these illustrate the far-reaching cardiac consequences of HDP and further demonstrate some of the racial inequities seen in outcomes.

Beyond the cardiovascular system, preeclampsia significantly impacts the kidneys and is the most common glomerular-based kidney disease in the world (37). In normal pregnancy, there is an increase in the glomerular filtration rate (GFR), resulting in a decrease in the serum creatinine value (38). Thus, normal creatinine values for a non-pregnant person may be pathologic in the state of pregnancy. Because of the altered physiology in pregnancy, a 30 to 40% reduction in GFR occurs prior to a significant elevation of the serum creatinine (39). Histologically, changes have been observed in the kidneys of people with preeclampsia, including endothelial swelling with a decrease in surface area for filtration as a result of increased subendothelial fibrinoid deposits (40). These changes contribute to an increased incidence of acute renal failure in pregnancy, with the rate in the United States increasing from 1.3 to 4.5 per 10,000 births between the years 1998 to 2008 (41).

These changes and renal pathologies can persist postpartum and are associated with significant maternal morbidity (42–44). One study followed patients for 1 year after childbirth, measuring estimated GFR at 3 and 12 months postpartum (45). This study illustrated a significant reduction in renal function as measured by estimated GFR at 3 and 12 months postpartum in patients after a pregnancy complicated by preeclampsia (45). Similarly, a large Norwegian study assessing risks for end-stage renal disease after pregnancy found that patients with a history of preeclampsia, after adjusting for confounders, continued to have an increased risk for the future development of end-stage renal disease (46). This risk increased with the number of pregnancies affected by preeclampsia (46). Compounding this association is the relationship between preeclampsia and the renal disease itself. Many risk factors for preeclampsia are also associated with the risk of developing renal disease later in life, so the true relationship may be difficult to determine fully. Regardless, patients with a history of preeclampsia have an increased risk of chronic renal disease later in life.

Blood cell dyscrasias and hepatic dysfunction are often observed in preeclampsia. Thrombocytopenia is observed in 30–50% of people with preeclampsia, with platelets less than 100 × 10^9^/L being diagnostic for preeclampsia with severe features (2). A combination of altered platelet clearance and hemolysis is thought to contribute to the thrombocytopenia seen in preeclampsia (47). Thrombocytopenia may also be caused from the activation and consumption of platelets caused by the endothelial injury seen in preeclampsia (48). Some studies have shown that certain platelet indices can be used for the prediction and early diagnosis of preeclampsia. However, this evidence is not conclusive and larger studies are needed (49). Hemolysis associated with preeclampsia is associated with an increased risk of poor outcomes, including acute kidney injury, blood transfusion, ICU admission, pulmonary edema, and poor neonatal outcomes (50). While the short-term implications on the hematologic system in patients with preeclampsia are relatively well understood, the long-term impact warrants further study.

Hepatic dysfunction in preeclampsia is marked by microvesicular fat changes and periportal and sinusoidal fibrin deposition in the liver parenchyma (51). These changes are typically transient and do not result in severe disability. Rarely (~1/40,000 to 1/250,000 pregnancies), in the context of preeclampsia, a subcapsular hematoma may form (52). This is potentially catastrophic, with resultant mortality rates ranging from 17 to 59% with an expanding hematoma or hepatic rupture (52). Rarely, patients may require a liver transplant after severe liver involvement in preeclampsia, though this is an exceedingly rare complication (52). With the exception of the more severe complications such as subcapsular hematoma, patients typically do not exhibit long-term hepatic impairment.

The nervous system is also significantly affected in pregnancy and preeclampsia. Eclamptic seizures are the most well-known neurologic sequela of preeclampsia, with an incidence of 0.5–1.5% of deliveries in developing countries but as low as 0.01–0.1% of deliveries in developed countries (53). Patients with eclampsia have an increased risk of disseminated intravascular coagulation, acute renal failure, pulmonary edema, heart failure, cerebrovascular disease, and death (53). Aside from eclampsia, there is an increased risk of a cerebrovascular accident caused by uncontrolled hypertension from preeclampsia (54, 55). Once patients are outside of the acute postpartum setting, their long-term risk of stroke remains elevated, with a two-fold increase in cerebrovascular accidents noted in patients with a history of HDP (56).

Some studies have suggested that patients with preeclampsia demonstrate long-term cognitive decline compared to those with pregnancies not impacted by preeclampsia (57). A retrospective study of 40 women at least 35 years from their antecedent pregnancy investigated neurocognition and dementia between patients who had pregnancies with hypertensive disorders of pregnancy and those without (57). Though not statistically significant, mild cognitive impairment was noted in a higher frequency in those with history of HDP compared to those without (p – 0.10) (57). Alternatively, some studies have questioned whether preeclampsia remains an independent risk factor for neurocognitive problems later in life. A large retrospective cohort study investigated the impact of a history of HDP on long-term cognition, illustrating that preeclampsia history was associated with decreased scores when measuring psychomotor function, memory, and executive function (58). This impact was no longer present once adjusted for age, BMI, education, depression, and hypertension (58). Though studies illustrate conflicting results regarding long-term cognitive impact, there is evidence to suggest that preeclampsia could, at the very least, contribute to neurocognitive dysfunction.

All complications of preeclampsia can worsen or occur for the first time in the immediate postpartum period, with hypertensive disorders of pregnancy being the leading cause of postpartum readmission (59). The postpartum period is an especially high-risk time, given the transition from at least weekly visits with a physician (antepartum) or continuous inpatient care (intrapartum) to no medical surveillance, usually until 6 weeks postpartum. Of the patients who are diagnosed with new, postpartum preeclampsia, 60% have never had a prior hypertensive diagnosis and present with severely elevated blood pressures and symptoms (60). As a result of this, people who are readmitted postpartum without a prior diagnosis of hypertension are at higher risk of eclampsia, stroke, and overall severe maternal mortality (61).

### 4.2. Fetal adverse outcomes

Though preeclampsia has significant sequelae in the pregnant person, there are also important implications for the fetus. As many as 1/3 of fetuses of patients with preeclampsia will develop fetal growth restriction (62). Fetal growth restriction itself carries an increased risk of stillbirth and neonatal death, necessitating increased healthcare visits and resultant costs as well as often inpatient admission (63). There is a seven-fold increase in the risk of intrauterine fetal death in preeclampsia with severe features as compared to pregnancies unaffected by hypertensive disorders (64). With these known risks, patients with preeclampsia are extensively monitored during their pregnancy. This monitoring consists of increased physician visits, laboratory evaluation, and fetal ultrasounds (2). Increased surveillance is more burdensome for the patient and costly for the healthcare system overall. If there is a high suspicion of worsening disease or a need for more monitoring, patients may be admitted for prolonged periods of time. Lastly, increased monitoring and the need for diagnostic evaluation can result in iatrogenic preterm delivery and the associated morbidities and costs that accompany preterm birth.

Newborns of pregnancies affected by preeclampsia are at higher risk of being small for gestational age and of having low seven-minute APGAR scores (65). Children born after pregnancies affected by preeclampsia are noted to have higher systolic blood pressures and body mass indices (66). A recent study demonstrated that there is persistent abnormal circulation in the offspring of patients with preeclampsia, including elevated pulmonary artery pressures (67). In addition, a few small studies have demonstrated changes in brain structural and vascular anatomy along with evidence of cognitive changes (67, 68). More studies are needed on the long-term outcomes of those born from pregnancies affected by hypertensive disorders of pregnancy.

## 5. Prediction of preeclampsia and related adverse outcomes

### 5.1. Antepartum

Many evidence-based screening guidelines have been developed in an attempt to diagnose preeclampsia and identify those at highest risk of adverse events. Numerous studies have illustrated that prophylactic treatment with low-dose aspirin therapy provides a significant decrease in the risk of preeclampsia (69). Traditionally, low-dose aspirin therapy has been reserved for these patients at high risk of HDP, with initiation as early as 12 weeks gestation. More recent evidence suggests that universal aspirin or higher dose aspirin therapy may be warranted (69). Various countries have different screening protocols to identify patients who will benefit from aspirin therapy. Current screening in the United States consists of the identification of major and moderate risk factors for preeclampsia development (Table 2) (70). Patients with one major and more than one moderate risk factor for preeclampsia are considered candidates for aspirin therapy. While aspirin therapy provides a significant reduction in preeclampsia development, more aggressive screening strategies and diagnostic modalities may aid in the increased reduction of disease.

**Table 2:** Clinical risk factors and risk stratification of patients.

Major Risk Factors
History of preeclampsia
Multifetal gestation
Chronic hypertension
Pregestational diabetes
Kidney disease
Autoimmune disease
Moderate Risk Factors
Nulliparity
Obesity
Family history of preeclampsia
Black race (representing systemic racism)
Lower income
>35 years of age
Personal history of low birth weight, previous adverse pregnancy outcome
>10 years between pregnancies
*In vitro* fertilization

Adapted from Davidson et al. (70).

Validated screening methods can not only aid in early risk stratification for patients who may benefit from aspirin therapy but can also provide information about who are likely to develop preeclampsia (71). The triple test, as released by the Fetal Medicine Foundation, utilizes uterine artery pulsatility, biomarkers (PlGF), and mean arterial pressure in the first trimester to predict those at the highest risk of disease development later in pregnancy with remarkable results (71). With this test alone, a 90% detection rate for early preeclampsia and a 75% detection rate for the preterm disease were achieved with a 10% false-positive rate (71). This strategy has been validated in various populations and represents a new and more specific way to screen for patients at high risk of disease development during pregnancy (71).

Similarly, the utilization of Pregnancy-Associated Plasma Protein (PAPP-A) has demonstrated another route through which screening for preeclampsia may be accomplished (72). A large prospective study in India assessed uterine artery pulsatility and maternal serum PAPP-A in predicting preeclampsia development. This study found that among patients with preeclampsia, PAPP-A levels on average were higher than those without the disease, with a sensitivity of 28%, specificity of 90%, and a detection rate of 79% (72). The negative predictive value of PAPP-A has been quoted to be as high as 97.55 with positive predictive value quoted at 2.95 (73). Uterine artery pulsatility was also significantly elevated in those who went on to develop preeclampsia, with a sensitivity of 68%, specificity 53, and 55% detection rate (72). The implementation of these types of first-trimester screening could further aid in the identification of patients who may derive the most benefit from prophylactic aspirin therapy or other antepartum intervention. Given the significant long-term health morbidity and mortality in those ultimately diagnosed with HDP, earlier detection to optimize prevention strategies represents an important way to improve health outcomes for patients.

In addition to early screening for preeclampsia prediction, current research focuses on strategies to predict those most likely to develop adverse outcomes related to their preeclampsia diagnosis (74, 75). Most of these studies focus on the exploitation of the proposed pathogenesis of HDP through the utilization of biomarkers to predict the development of worsening preeclampsia and adverse outcomes. As mentioned earlier in this review, sFlt-1 and PIGF are two biomarkers that have been implicated in the development of preeclampsia and have also been investigated as predictors for adverse outcomes related to preeclampsia (74). Studies have shown that levels of these markers, and specifically the ratio of sFlt-1/PIGF, are different in pregnancies affected by HDP (74, 76). Furthermore, these biomarkers are altered more significantly in patients with early-onset preeclampsia, and those with the early-onset disease generally have more severe long-term sequelae (75).

Given that preeclampsia with severe features in general results in more significant morbidity compared to lesser HDP, utilizing biomarkers may help aid clinicians in triaging patients to more aggressive therapy and monitoring or outpatient management. A cohort study in the United States investigated the predictive value of sFlt-1/PlGF among patients presenting to obstetrical triage for preeclampsia evaluation (77). A ratio of >38 in patients with suspected preeclampsia and patients diagnosed with preeclampsia without severe features while in triage was predictive of the development of preeclampsia with severe features within 2 weeks of presentation (OR 15.6%, confidence interval 8.91–27.40 for restrictive diagnosis, and OR 14.56% with 95% confidence interval 8.30–25.56 for broader diagnosis) (77). Similarly, another large study assessed the value of sFlt-1/PlGF in predicting progression to preeclampsia with severe features and identifying those at the highest risk of adverse maternal outcomes (78). In patients between gestational ages of 23 and 35 weeks, a ratio of >40 (PPV 65% [95% CI 59, 71] and NPV 95% [95% CI 93, 98]) similarly showed an increased risk in progression to severe disease, but also an increased risk in adverse maternal outcomes (78). Blood pressures alone in the antepartum and intrapartum period have a poor positive predictive value (PPV) for the accurate prediction of adverse outcomes (PPV 18–20% with antepartum and intrapartum blood pressures and 22–36% with antepartum blood pressures alone) (79). The development of machine-based learning models has also shown promise in identifying early- and late-onset preeclampsia as well as those at the highest risk of adverse outcomes (80–82); however, it is not readily available for clinical use. The integration of early screening for preeclampsia and biomarker use to aid in the determination of those at the highest risk of disease progression and adverse outcomes is becoming an important tool to improve the healthcare of pregnant persons suffering from preeclampsia.

### 5.2. Postpartum

Patients in the postpartum period remain at risk for development of HDP in the postpartum period and long-term sequelae related to a prior diagnosis of preeclampsia. In the postpartum period, there are tools that exist to aid in minimizing these adverse outcomes. Studies have illustrated that postpartum blood pressure monitoring in patients with chronic hypertension and HDP is a sustainable and important intervention for patients in the postpartum setting (83, 84). One tertiary care center implemented a postpartum blood pressure monitoring program that included standardized education and assisted follow-up, illustrating a dramatic increase in postpartum visit attendance (33.5% vs. 59.4%, *p* < 0.001) with more patients reporting blood pressures of <140/90 (39.1% vs. 18.5%, *p* = 0.004) (84). Importantly, when incorporating telemedicine, racial disparities in visit compliance were reduced, providing one mechanism through which health equity in the setting of postpartum care can be mitigated (85). Utilizing programs such as these might provide an important mechanism through which postpartum adverse outcomes can be prevented.

The American Heart Association recognizes that adverse pregnancy outcomes (APO’s), including HDP, increase the risk of cardiac disease for the pregnant person (86). Though how exactly to incorporate a history of HDP in a formal risk evaluation is not clearly established, this organization recommends that providers caring for patients with a history of HDP recognize this as an important risk factor for future disease and place a strong emphasis on primary prevention of cardiac disease in these patients (86). Recommendations include heart healthy diet, maintaining an appropriate weight, and engaging in recommended amounts of physical activity (86). Even though clear guidelines do not exist regarding how to modify a patient’s cardiovascular risk score with this history, the importance of recognition and close follow-up cannot be understated to help prevent long-term morbidity.

## 6. Conclusion

Preeclampsia and hypertensive disorders of pregnancy are diagnoses that contribute significantly to maternal and fetal morbidity and mortality. A better understanding of how to prevent and treat these disorders is crucial to improving maternal and fetal/child health. An accurate understanding of the development of preeclampsia and disease progression represents an important tenant in improving patient care and preventing adverse outcomes. In addition, future treatment modalities targeting known pathogenic mechanisms are an area ripe for future studies. Patients with preeclampsia carry an increased risk of major morbidity and mortality throughout their life beyond the immediate postpartum period, underscoring the importance of prevention and treatment of this disease. Preeclampsia has a significant impact on overall cardiac health and the development of future cardiovascular disease, development of chronic hypertension, hepatic and hematopoietic dysfunction, and renal and neurologic outcomes. This disease has important immediate and long-term implications for the neonate, further illustrating the importance of accurate treatment and prevention of preeclampsia. Given the enormity of this impact on short-term and long-term health, more research into prevention is needed along with an emphasis on long-term follow-up after a pregnancy complicated by HDP.

## Author contributions

SR initiated the study conception and design. CB and SD wrote the initial drafts of the manuscript. EP and SS collected the references and edited the manuscript. PD made the figures for the manuscript and edited the manuscript. All authors have contributed to the writing and editing of the manuscript and reviewed and approved the submitted version.

## Conflict of interest

SR reports serving as a consultant to Roche Diagnostics and Thermo Fisher Scientific and has received funding from Roche Diagnostics and Siemens for studies related to the use of angiogenic factors in pregnancy which is unrelated to work for this manuscript.

The remaining authors declare that the research was conducted in the absence of any commercial or financial relationships that could be construed as a potential conflict of interest.

## Publisher’s note

All claims expressed in this article are solely those of the authors and do not necessarily represent those of their affiliated organizations, or those of the publisher, the editors and the reviewers. Any product that may be evaluated in this article, or claim that may be made by its manufacturer, is not guaranteed or endorsed by the publisher.
